# Primary umbilical endometriosis: a case report

**Published:** 2017-06

**Authors:** DPA Van den Nouland, M Kaur

**Affiliations:** Department of Gynaecology and Obstetrics, Zuyderland Medisch Centrum, Sittard-Geleen, The Netherlands.

**Keywords:** Cutaneous endometriosis, primary umbilical endometriosis, extragenital endometriosis, diagnosis, treatment

## Abstract

Primary umbilical endometriosis is a rare phenomenon accounting for 0,4-1,0% of extragenital endometriosis. Despite the fact that it mostly presents as a typical cyclic umbilical discharge coincidental with a palpable mass, the diagnosis is often delayed due to its low prevalence, as was seen in the presented case. The exact pathogenesis is still unclear. The golden standard for diagnosis is histopathological examination, but diagnostic tools like ultrasound, MRI or CT scan can be helpful. The differential diagnosis includes a wide range of disorders. Surgical management is the preferred treatment.

## Introduction

First described by Rokitansky in 1860, endometriosis is characterised by the presence of endometrial tissue outside of the uterine cavity (Giudice and Kao, 2004; Pramanik et al., 2014). It is a benign, oestrogen-dependent chronic disorder, with a prevalence of 6% - 15%. The vast majority of symptomatic women experience dysmenorrhoea, dyspareunia and/or deep pelvic pain, whereas 30% - 50% deal with subfertility (Hirsch and Davis, 2015; Boesgaard-Kjer et al., 2016). However, symptoms vary depending on the site and severity of the endometriosis related in ammation. Implants have been reported in the pericardium, pleura, liver, and even in the brain (Giudice and Kao, 2004; Bulun, 2009; Boesgaard-Kjer et al., 2016).

Cutaneous endometriosis is much less common and is mostly caused by iatrogenic seeding of endometrium during pelvic surgeries, resulting in endometriosis implants in the abdominal scars. Primary umbilical endometriosis (PUE) is a rare subtype of spontaneous occurring cutaneous endometriosis and is estimated to account for only 0,5% to 1% of all extragenital endometriosis (Ghosh and Das, 2014; Boesgaard-Kjer et al., 2016; Taniguchi et al., 2016). In primary cutaneous presentation, umbilical endometriosis accounts for up to 40% of the cases. Although several theories postulate the ambiguous origin of endometriosis - including retrograde menstruation, iatrogenic spread, embryonic cell rest, coelomic metaplasia and, haematogenous or lymphogenic spread – its pathogenesis has remained elusive. Likewise, the pathogenesis of PUE is also still unclear. The suggested possible theories in PUE are composed of endometrial cells migrating to the umbilicus through the abdomen or the lymphatic system, and/ or remnants of embryonic cells in the umbilical fold (Victory et al., 2007).

We present a case of a PUE without any suspicion of concomitant pelvic endometriosis, supported with insights from the state of the art literature on this rare endometriosis entity.

## Case report

A 44 year old Caucasian woman was referred by her general practitioner, with complains of a painless, intermittent dark red to brownish coloured discharge from her umbilicus, which was becoming bloody in appearance since a few months. She had started to notice that this discharge was consistently starting just prior to her menstruation. These symptoms were intermittently present for about 1 year. She had been prescribed topical treatments with anti-fungal and/or antibacterial properties several times by her general practitioner, with no improvement. She had an unremarkable medical history, without any surgeries, abdominal trauma or subfertility, and had one normal vaginal delivery. There were no complaints of dysmenorrhoea, dyspareunia, pelvic pain or dyschezia. She had used oral contraceptive pills in the past, but stopped using these approximately 14 months ago, because her husband underwent vasectomy.

**Figure 1:**
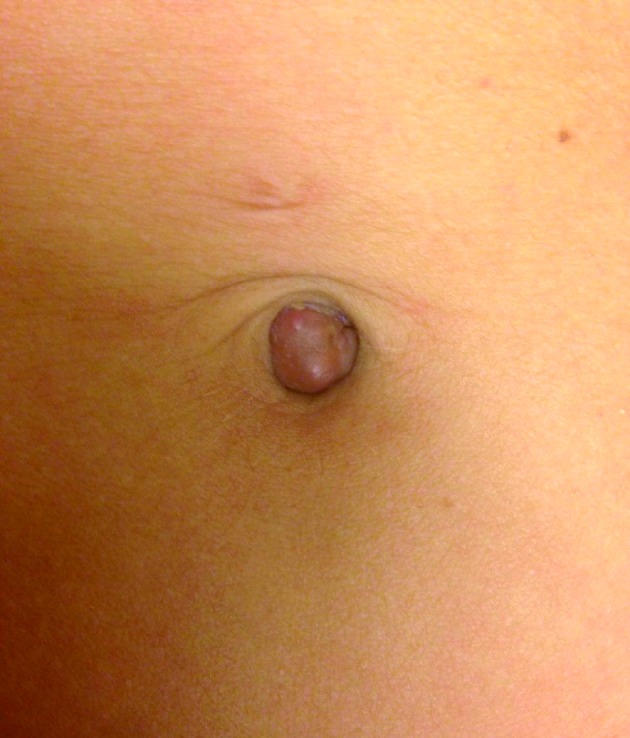
— A superficial hyperpigmented dark reddish to violaceous nodule, located in the umbilicus of approximately 1 cm.

**Figure 2:**
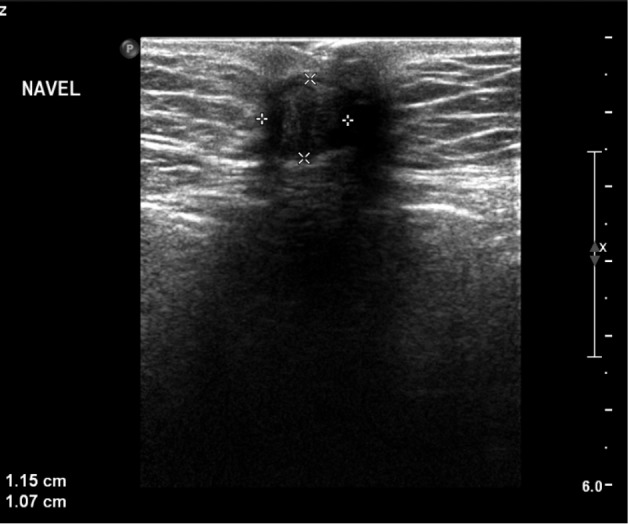
— The abdominal ultrasound, showing a superficial nodule of 1.15 to 1.07cm in the umbilicus.

Physical examination revealed a superficial hyperpigmented dark reddish to violaceous nodule, located in the umbilicus of approximately 1 cm (Fig. 1). The nodule was not painful at palpation, was irreducible by gentle pressure and the patient stated that its size underwent periodic changes. Ultrasound (US) of the abdominal wall showed a superficial nodule of 1.15 x 1.07 cm, with no invasion of the abdominal fascia (Fig. 2). No increased angiogenesis was seen. Although a definitive diagnosis could not be obtained, under the presumptive diagnosis of cutaneous umbilical endometriosis, a local excision of the umbilical lesion was planned. Based on the reported efficacy of gonadotropin- releasing hormone (GnRH) analogues in pelvic endometriosis, a two-month treatment with GnRH analogues was given to our patient preoperatively, in order to have symptomatic relief and to evaluate whether this could promote a size reduction of the umbilical lesion.

Following this, an omphalectomy was executed under general anaesthesia, hereby excising the umbilicus en bloc with the nodule; the incision was made lateral to the umbilicus while a progressive dissection around the nodule was carried out until normal tissue was visible in the margins. The excised specimen resulted in approximately 2 cm diameter of tissue, mainly due to fibrotic tissue around the endometriotic nodule. The abdominal fascia remained intact. A plastic surgeon performed the closure of the umbilicus by simple interrupted non-absorbable sutures.

**Figure 3:**
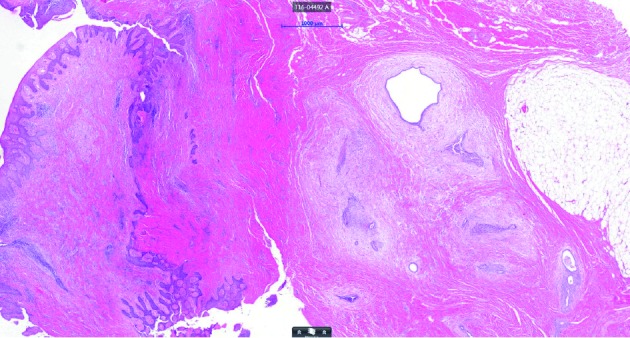
— Fibro-adipose connective tissue with prevalence of multiple endometrial glandular tubes and surrounding endometrial stroma..

Histopathological examination of the excised tissue revealed fibro-adipose connective tissue with widespread prevalence of multiple endometrial glandular tubes and surrounding endometrial stroma (Fig. 3). There were no signs of endometrial hyperplasia, atypia or malignancy. Concluding, the findings were compatible with diagnosis of umbilical endometriosis.

**Figure 4:**
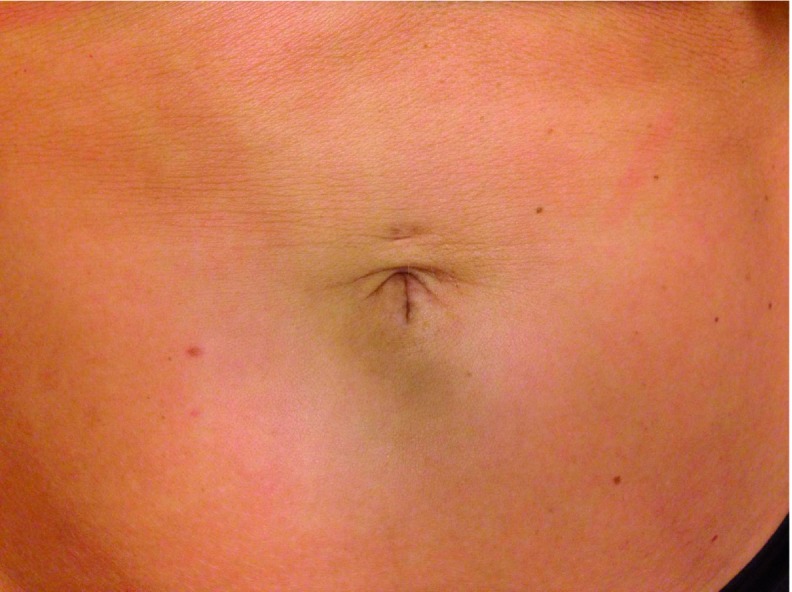
— Postoperative umbilical scar

Six days post-surgery, the patient presented with periumbilical pain. Clinical findings were suggestive for local inflammation at the wound. A bacterial culture was positive for Staphylococcus epidermidis and bacteroides pyogenes. The patient was given antibiotics for 7 days after which she had a normal recovery and was very satisfied with the final stage of postoperative umbilical scar (Fig. 4).

## Discussion

Extragenital endometriosis is seen in 1% - 12% of all patients diagnosed with endometriosis (Victory et al., 2007; Kahlenberg and Laskey, 2014). PUE is also known as Villar’s nodule, with reference to the author who described in 1886. The most common site involved in spontaneous (primary) cutaneous endometriosis is the umbilicus, hypothetically acting as a physiologic scar and having a predilection for ectopic endometrial implants. Mean age at the time of PUE diagnosis is estimated at 35-38 years (Giudice and Kao, 2004; Victory et al., 2007) reflecting that the manifestation of this disorder occurs after being activated by an extended exposure to hormonal-, metaplastic- and/or environmental factors. Till date, there has been only one case reported of umbilical endometriosis in a postmenopausal woman (58 years) which was in addition, coexistent with metastatic endometrial adenocarcinoma of the umbilicus (Ikeda et al., 2006), solidifying PUE’s oestrogen-dependant susceptibility. There is a long interval from the onset of symptoms to the final treatment, with an estimated mean of 13.3 months in the literature (Victory et al., 2007). In our case, both the mean age at onset and this interval are relatively consistent with these findings.

The most commonly accepted theories to elucidate the pathogenesis of endometriosis are the ‘coelomic metaplasia theory’ and ‘the retrograde menstruation theory’. While the coelomic metaplasia theory suggests a differentiation or transformation of peritoneal mesothelial cells (with an embryonic coelomic origin) and/or mullerian remnants into endometrial tissue in the peritoneal cavity, the latter theory proposes the spread of endometrial tissue by retrograde menstruation and implantation of these cells into the pelvic structures (Bulun, 2009; Calagna et al., 2015). In addition, some other theories postulating the origin of endometriosis have been described. In the lymphatic or haematogenous spreading, the endometrial cells enter the uterine circulation and can reach different organs at distant sites (Koninckx et al., 2000; Taniguchi et al., 2016). The role of host factors with evidence for an altered immune system function in patients with endometriosis is proposed by some, as this has been reported to be measurable in peritoneal fluid and serum, hereby showing increased cytokine and prostaglandin production, increased B cell activity and decreased natural killer cell activity (Dmowski et al., 2004; Cakmak et al., 2009). The potential role of genetic predisposition has been hypothesized as well (Bischoff and Simpson, 2000). In PUE with concomitant pelvic endometriosis, local inflammation around ectopic implants may support shedding of endometrial cells followed by their migration to the umbilicus, while in isolated PUE the mechanism of metaplastic changes in the urachal remnant has been described (Mizutani et al., 2012).

As seen in our case, the typical presentation of PUE is a bluish/purple mass, accompanied with catamenial bleeding from the umbilicus with or without associated pain or tenderness (Taniguchi et al., 2016). Besides, the condition can be completely asymptomatic in some cases, while there are also cases reporting continuous pain (Victory et al., 2007; Calagna et al., 2015). Since 1990, less than 100 cases of PUE have been reported, most of them illustrated in single case reports. The size of PUE ranges from 0,5 to 3 cm in the most reported cases, with an average of 2.3 cm (Victory et al., 2007). Histological confirmation is the current golden standard for diagnosing PUE, while initial diagnosis for treatment workup is primarily clinical. Diagnostic tools like transcutaneous US, MRI or CT scan can be helpful to investigate the relationship of the nodule with the surrounding tissue and to differentiate between other umbilical lesions, for example umbilical hernia (Ghosh and Das, 2014; Pariza and Mavrodin, 2014; Boesgaard-Kjer et al., 2016). Fine needle aspiration cytology can be supplementary, but inconclusive results have been reported to be as high as 75% (Victory et al., 2007; Kimball et al., 2008). Furthermore, elevated levels of CEA and CA125 tumour markers may raise the suspicion of concomitant pelvic endometriosis lesions (Calagna et al., 2015). In our case, the typical presentation along with an US revealing no invasion in the underlying structures was found sufficient to establish a tentative diagnosis in order to initiate treatment.

Histological findings are characterised by irregular endometrial glandular structures in basophilic cytoplasm, accompanied by high cellular and vascular stroma, which have a spindle-cell appearance. Cutaneous endometriotic lesions can show a mixture of different menstrual phases within the same lesion, just as in endometrial sampling of women with dysfunctional bleeding. The presence of hemosiderin deposits in the stroma is a common finding, whereas inflammation and marked mitotic activity can be seen as well. A careful assessment should be made to rule out atypia to exclude malignancy, especially if hypertrophic or myxoid decidual changes appear. Keratin 7+/ keratin 20- expression, along with expression of ER, PR receptors and Ki-67, is in accordance with the immunohistochemical properties of normal endometrium. Metastatic adenocarcinoma from the digestive system is typically keratin-/keratin 20+. CD10 has also been reported to useful in establishing an unclear diagnosis, as this is strongly expressed in endometrial stromal nodules, however, its usefulness has been speculated by some due to the fact that several fibroblasts of the normal dermis express this neutral endopeptidase. Additionally, calretinin expression is low to absent in ectopic endometrium, whilst it is expressed in eutopic endometrium (Farooq et al., 2011; Kyamidis et al., 2011). The histology in our case showed similar characteristics of endometrial glandular- and stromal tissue without atypia, however, expression of keratin 7, ER- and PR receptors was not assessed.

The differential diagnoses include melanocytic naevus, endosalpingiosis presenting as periumbilical papules, pyogenic/foreign body granuloma, umbilical polyp, seborrheic keratosis, epithelial inclusion cyst, desmoid tumour, haemangioma, granular cell tumour, umbilical hernia, omphalitis and keloid (Malebranche and Bush, 2010; Ghosh and Das, 2014). Primary or secondary metastatic neoplasms, such as melanoma or Sister Mary Joseph’s nodule, should be ruled out (Victory et al., 2007; Ghosh and Das, 2014; Pariza and Mavrodin, 2014). Nevertheless, the risk of malignancy is considerably low. Only three cases of umbilical endometriosis associated with malignancy have been reported: - first showing a malignant transformation, more than forty years after first episode of catamenial umbilical bleeding at the age of 30 years which occurred until menopause (Lauslahti, 1972), - second as above mentioned, coexisting with metastatic endometrial adenocarcinoma of the umbilicus (Ikeda et al., 2006), and - third was recently reported as a clear cell carcinoma transformation from umbilical endometriosis (Obata et al., 2013).

Surgical management is the preferred and definitive treatment modality, involving a local excision (respecting margins of 1cm) with/without a resection and repair of the underlying fascia according to the depth of the lesion (Purvis and Tyring, 1994; Paramythiotis et al., 2014; Chikazawa et al., 2014). The literature reports 13%-15% incidence of simultaneous pelvic endometriosis presence (Chikazawa et al., 2014). As such, although some authors suggest a concurrent laparoscopic pelvic evaluation, this combined approach is not obligatory but should be considered in cases, which present with a high index of suspicion for pelvic endometriosis (Victory et al., 2007; Calagna et al., 2015; Boesgaard-Kjer et al., 2016). Medical treatment with oral contraceptives, progesterone, Danazol or GnRH analogues is still under debate. The literature reports their utilization with the intention to ameliorate the symptoms by reducing the size of the nodule, hereby also limiting the amount of specimen to be excised and reducing angiogenesis. However, the overall results seem to be inconsistent and poor, which may be explained by the relatively low levels of oestrogen receptors found in cutaneous endometriosis lesions (Victory et al., 2007; Ghosh and Das, 2014; Calagna et al., 2015; Boesgaard-Kjer et al., 2016; Kydd et al., 2016; Taniguchi et al., 2016). In accordance with these findings, although our patient became asymptomatic when receiving pre-treatment with GnRH analogue, there was no noticeable reduction encountered of the umbilical nodule. The prognosis of PUE is good with a low recurrence rate following an optimal and complete surgical excision.

## Conclusion

PUE is a relatively rare sub-type of endometriosis, leading to a delayed presentation and/or referral despite the fact that in most cases a macroscopic visible lesion is present. Although the pathogenesis of PUE is not completely understood, considering the current increased focus on diagnosing and managing endometriosis, the prevalence of PUE can show a potential rise and this elucidates the importance of correctly identifying PUE, which is primarily a clinical diagnosis, followed by a histopathological confirmation. Because surgical management is the mainstay modality of treatment, an early diagnosis avoids an overly extensive local excision besides giving symptomatic relief. Practitioners of different specialties should consider PUE when encountering an umbilical mass in a woman during physical examination.
